# Simultaneous functional near-infrared spectroscopy and electroencephalography for monitoring of human brain activity and oxygenation: a review

**DOI:** 10.1117/1.NPh.4.4.041411

**Published:** 2017-08-22

**Authors:** Antonio M. Chiarelli, Filippo Zappasodi, Francesco Di Pompeo, Arcangelo Merla

**Affiliations:** a University of Illinois at Urbana Champaign, Beckman Institute, Urbana, Illinois, United States; b Università G. d’Annunzio, Department of Neuroscience, Imaging and Clinical Science, Chieti, Italy; c Università G. d’Annunzio, Institute for Advanced Biomedical Technologies, Chieti, Italy

**Keywords:** functional near-infrared spectroscopy, electroencephalography, multimodal monitoring, noninvasive brain imaging, flexible brain imaging, neurovascular coupling

## Abstract

Multimodal monitoring has become particularly common in the study of human brain function. In this context, combined, synchronous measurements of functional near-infrared spectroscopy (fNIRS) and electroencephalography (EEG) are getting increased interest. Because of the absence of electro-optical interference, it is quite simple to integrate these two noninvasive recording procedures of brain activity. fNIRS and EEG are both scalp-located procedures. fNIRS estimates brain hemodynamic fluctuations relying on spectroscopic measurements, whereas EEG captures the macroscopic temporal dynamics of brain electrical activity through passive voltages evaluations. The “orthogonal” neurophysiological information provided by the two technologies and the increasing interest in the neurovascular coupling phenomenon further encourage their integration. This review provides, together with an introduction regarding the principles and future directions of the two technologies, an evaluation of major clinical and nonclinical applications of this flexible, low-cost combination of neuroimaging modalities. fNIRS–EEG systems exploit the ability of the two technologies to be conducted in an environment or experimental setting and/or on subjects that are generally not suited for other neuroimaging modalities, such as functional magnetic resonance imaging, positron emission tomography, and magnetoencephalography. fNIRS–EEG brain monitoring settles itself as a useful multimodal tool for brain electrical and hemodynamic activity investigation.

## 1 Introduction

Investigation of human brain functions has become of great interest within the scientific community. Because of the multiple physiological information that brain activity can provide, several techniques have been developed over the years to study brain signals coming from different neurophysiological mechanisms. Due to the absence of a specific technology that can record the whole spectrum of the information generated by these signals, simultaneous multimodal monitoring of brain state has become increasingly common in the last decade. Among multimodal monitoring, the integration of functional near-infrared spectroscopy (fNIRS) and electroencephalography (EEG) is receiving increased interest.

fNIRS is a relatively new neuroimaging technique that has become a useful tool for brain activity monitoring due to its portability and lightweight properties and its limited costs. fNIRS is a scalp-based optical spectroscopic measurement that uses light injection and detection points to measure hemodynamic fluctuations in the brain tissues.^1^^–^^3^ fNIRS can record the blood oxygen level dependent (BOLD) effect, which is the compensatory hemodynamic response occurring in the brain due to the increased oxygen demand in activated brain areas. fNIRS relies on differential measurements of the backscattered light, which is sensitive to changes in concentration of the two principal oscillating absorbing chromophores in the near-infrared (NIR) spectral range: oxy- and deoxyhemoglobin ($O_{2}\mathrm{Hb}$ and HHb). $O_{2}\mathrm{Hb}$ and HHb have different absorption spectra in the NIR range (wavelengths between 650 and 900 nm).^1^^,^^4^ This characteristic, coupled with the water’s low absorption within the same wavelength range, allows the measuring of relative concentration and oscillations of these substances. Over the years, fNIRS technology has become a widely applied brain imaging method in different populations and experimental conditions.^5^^–^^15^

EEG is a well-established technique in neurological and neuroimaging settings^16^ suitable for capturing the macroscopic temporal dynamics of brain electrical activity through the passive measurements of scalp located voltages. EEG systems are widely used for clinical and nonclinical purposes to diagnose and monitor brain functions and dysfunctions.^17^

Electrical brain activity and its hemodynamic counterpart do not have a perfect spatiotemporal correspondence.^18^ Their interaction is mediated through the neurovascular coupling mechanism that can be studied using the combined technologies. On the contrary, if a model of the neurovascular coupling is assumed, increased accuracy on the neural signal estimates can be obtained from multimodal measurements.^19^^,^^20^ The two recording procedures share multiple advantages: fNIRS and EEG technologies are decently robust against motion artifacts and do not impose remarkable physical constraints, particularly when compared with functional magnetic resonance imaging (fMRI), positron emission tomography (PET), or magnetoencephalography (MEG), thus being feasible for more natural types of cognitive tasks and for a wide range of populations (e.g., from infants to elderly people). Moreover, fNIRS and EEG do not involve exposure to high-intensity (>1  T) magnetic fields nor ionizing radiations. Last but not less important, hardware costs are significantly lower than those of most other functional brain imaging modalities (fMRI, PET, and EEG).

This review is structured as follows: the first section is dedicated to the description of the physical principles, the origins, and the evolutions of both fNIRS and EEG. The second section is dedicated to analyzing the main advantages in the coupling of the two techniques and to investigating the major nonclinical and clinical applications of fNIRS–EEG measurements. Finally, the last section is devoted to underlining the limitations, challenges, and future direction of fNIRS–EEG integration. A conclusion section ends the review. It should be stressed that, with respect to the application section, the goal of the review is to report to the reader an up-to-date overview of where and for what purposes the multimodal procedure was applied, and it is not intended to report meta-analysis or to perform an in-depth critical evaluation of the specific findings within each application. In fact, these analyses would be not suited for a single, broad-topic review of the technology and its applications and should be reported in more focused, single-field-of-application, reviews.

## 2 Technology, Principles, and Evolution

### 2.1 Functional Near-Infrared Spectroscopy

**Fig. 1:**
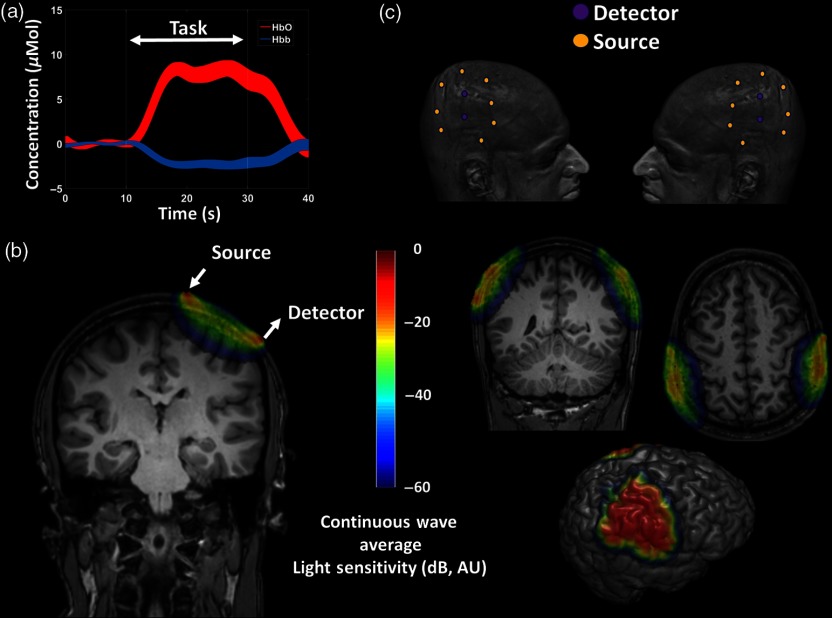
(a) Example of a typical “BOLD” response recorded by fNIRS in a task-activated brain region. Average changes in HHb and $O_{2}\mathrm{Hb}$ concentrations are reported together with their variability (standard error) for each time point. The BOLD response in active brain areas is characterized by an overcompensatory supply of $O_{2}\mathrm{Hb}$ with a concurrent wash-out of HHb, typically with a ratio ($O_{2}\mathrm{Hb}/\mathrm{HHb}$) of ∼3 and consisting of few μmol changes. (b) Coronal head slice of a typical continuous wave light-sensitivity pattern (logarithmic scale) for a source–detector couple positioned on the scalp (fNIRS channel), overlaid on a structural MRI. The light-sensitivity pattern was computed using a finite element method approach.^21^ (c) Example of a possible optical array and channels’ average light sensitivity pattern (logarithmic scale, averaged across multiple channels), employed to the imaging of motor and sensorimotor cortices, overlaid on a subject structural MRI and extracted gray matter. Multiple sources and detectors are required to increase the “field of view” of the fNIRS technology.

The most common application of NIR light in the study of the human brain is fNIRS. fNIRS measures changes in the optical properties of brain tissue in the NIR range (650 to 950 nm) to estimate fluctuations in the concentration of HHb and $O_{2}\mathrm{Hb}$ associated with neural activity. In fact, HHb and $O_{2}\mathrm{Hb}$ are the main time-varying chromophores in this spectral range, and they provide different absorption spectra.^1^^,^^4^ This characteristic, coupled with the water’s low absorption and the high tissue diffusive properties within the same wavelength range, makes it possible to measure relative concentrations of these substances directly from the scalp. Based on hemoglobin fluctuation measurements, fNIRS can record the BOLD effect, which is the compensatory hemodynamic response occurring in the brain due to the increased oxygen demand in activated brain areas. Because of the low-frequency characteristic of the BOLD response (<0.1  Hz), the fNIRS signal has intrinsic low temporal resolution capabilities. Whereas a functional MRI measures the BOLD effect primarily because of its sensitivity to the HHb and its paramagnetic properties, fNIRS can uncouple the HHb and $O_{2}\mathrm{Hb}$ fluctuations present in the BOLD response [Fig. 1(a)]. An estimate of total hemoglobin changes (HbT) related to cerebral blood volume (CBV) changes may be further computed.

Jöbsis^22^ reported that brain tissue transparency to NIR light allowed a noninvasive and continuous monitoring of tissue oxygen using forward-scattering measurement (transillumination). Transillumination was mainly used in children, and it was of limited utility for studying adult brain; it was replaced by the backscattering mode procedures. Development of fNIRS systems proceeded rapidly, and, by the mid-1980s, the first studies on cerebral oxygenation using a backscattering approach were conducted.^23^

Standard fNIRS measurements rely on injecting light (using light sources with power of ∼1  mW), and detecting it in a backscattering geometry (through highly sensitive light detectors, up to single photon sensitivity capabilities), the coupling of which (channel) creates a typical sensitivity pattern in the highly diffusive head structures [Fig. 1(b)]. fNIRS typically rely on injecting and detecting light in and from the scalp through optical fibers/fiber bundles. Optical fibers electrically isolate the subject, and they allow light sources and detectors to be located sufficiently far from the scalp. Different instrumental technologies procedures have been developed and used for fNIRS monitoring. Three main classes can be identified: time domain (TD),^24^ frequency domain (FD),^25^ and continuous wave (CW)^26^ recording systems. TD systems use very short pulses of light (picoseconds) whereas FD systems use light modulated at radio frequencies (>50  MHz) to investigate the tissue of interest. CW systems, because of the simple technological characteristics, are the most diffused systems both in clinical and nonclinical settings. CW systems rely on measuring the CW component of the light that traveled through the tissue. They can provide estimates of hemoglobin and oxygenation changes over time (by employing the modified Beer–Lambert equation with *a priori* differential pathlength factors^27^), but they do not provide absolute estimation of tissue optical properties (absorption and reduced scattering coefficient) at a channel level. They can measure regional cerebral oxygen saturation (${\mathrm{rSO}}_{2}$) and fractional tissue oxygenation extraction (FTOE) variability over time when a spatially resolved spectroscopy approach is employed.^28^

In this review, we focus on CW measurements and their application to combined fNIRS–EEG. When we further refer to and/or describe fNIRS throughout the paper, we mean CW-fNIRS, unless explicit reference to TD or FD systems is made.

Because light sensitivity decays exponentially (within a few cm) from the given source–detector couple, fNIRS provides very good localization power parallel to the scalp surface. However, it requires multiple optodes (sources and detectors) to properly cover the region of interest [Fig. 1(c)]. A problem that affects fNIRS measurement is its high sensitivity to scalp-related (extracerebral) hemoglobin oscillations. However, this confounding factor can be overcome by investigating multiple source–detector distance measurements. fNIRS provides different depth sensitivities depending on the source–detector distance employed. Generally, in adults, distances of around 3 cm or more are sensitive to both extracerebral and brain-related hemodynamic fluctuations. On the contrary, short distances (around 1.5 cm) are sensitive only to extracerebral hemodynamic components,^29^ since their sensitivity patterns do not reach the brain cortex. This characteristic allows the creation of topographic images of brain activity using longer distance channels or a combination of longer and shorter distances. Channel-based topographic fNIRS imaging can reach a good depth sensitivity (around 3 cm from the scalp using longer source–detector distances); however, depth localization of hemoglobin fluctuation is limited.

Tomographic reconstruction of hemoglobin oscillations can be achieved through fNIRS [diffuse optical tomography (DOT)] by exploiting the different light-sensitivity patterns of different source–detector distances.^30^^,^^31^ Using high-density optical arrays and combining multiple source–detector distances (from around 1.5 to 6 cm), good tomographic images can be obtained up to 3 cm from the scalp with localization power of ∼2  mm and spatial resolution of ∼1.5  cm (HD-DOT).^32^^–^^34^ However, the high density of optodes required for HD-DOT makes the instrumentation expensive and, because of the presence of many optodes, hard to be implemented with concurrent EEG recordings. Thus, in combined fNIRS–EEG measurements, sparse optical arrays are generally implemented.

fNIRS measurements and the corresponding signal processing techniques have evolved over time, and they share common features with the well-established analysis of fMRI signals.^35^ Blocked and event-related designs as well as resting state experiments may be employed. Signal analysis over time involves, among other things: motion artifact correction, filtering, averaging, deconvolution, general linear model (GLM), principal component analysis (PCA), and independent component analysis. Signal analysis over space and the related statistical procedures generally rely on filtering, PCA, spatial clustering, and false discovery rate controlling procedures (Gaussian random field theory, etc.).^35^^–^^37^

### 2.2 Electroencephalography

Since the first recording of the human brain electric potential on the scalp in 1924, EEG has been widely developed and has become the cheapest, fastest, and most diffuse noninvasive method to monitor brain electrophysiology, particularly in clinical environments (for an extensive review on EEG and its clinical application, see Ref. 38). EEG provides a very high temporal resolution of brain activity (∼1  ms), allowing the tracking of the cerebral dynamics with the temporal detail of the neuronal processes.

**Fig. 2:**
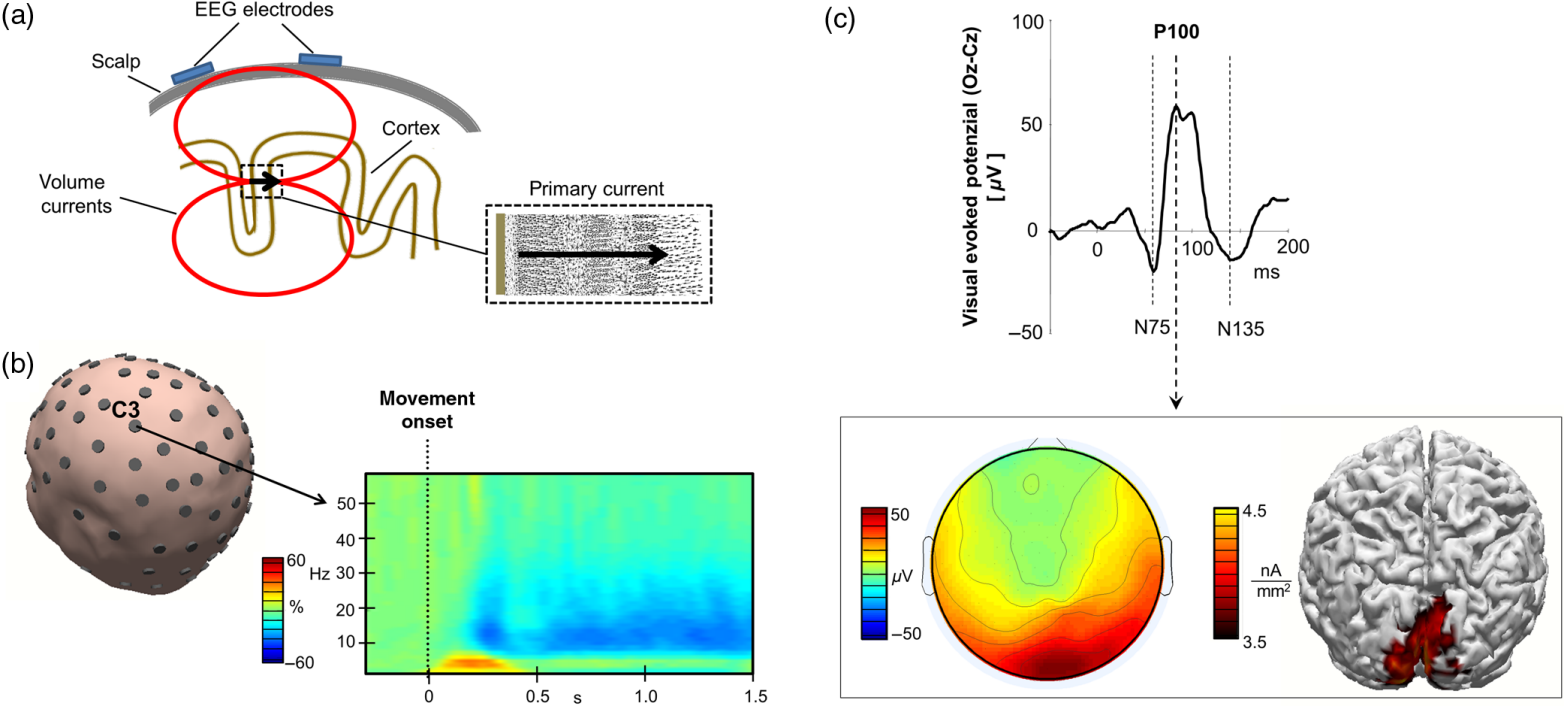
(a) Schematic representation of the EEG signal generation. The synchronous activity of a large number of neurons generates electric fields that, if synchronous, can add up to produce a signal intense enough to be detectable by electrodes placed on the scalp. The primary currents are mainly the result of the synaptic potentials in correspondence of the dendritic trees, which follow a preferential direction, as in the case of the pyramidal neurons. The neurons are surrounded by the cerebral tissues, i.e., a conductive medium. The primary current provokes extracellular currents flowing thorough this medium, as well as through the cerebrospinal fluid, the skull, and the scalp. These currents, named secondary or volume currents, reach the scalp and generate voltage differences, detectable by a pair of EEG electrodes. (b) Example of time-frequency representation of EEG signal at C3 (located over the left motor cortex) during a visually guided finger tapping task, executed with the right hand. For each frequency, the power is expressed as percentage variation of the corresponding value in the premovement period, evidencing a reduction in the alpha and beta rhythms during the movement (ERD) and an increase of theta rhythm within the first 500 ms from the movement onset (ERS). (c) Example of VEP obtained by a pattern-reversal stimulation. The EEG activity was recorded by a 128-channel system (EGI). Top: average response locked to the stimulus for the electrode Oz (referred to Cz), placed on the occipital lobe in correspondence to the visual cortex. The VEP consists of a sequence of negative-positive-negative peaks at specific latencies. The parameters that are considered to describe these waves are the latency and the amplitude of such peaks, which are referred to as N (negative) or P (positive) depending on the polarity with respect to a specific montage. In the case of the VEP, N75-, P100-, and N135-components can be seen. Bottom: on the left the topographical map shows the interpolation above the scalp surface of the values of all EEG sensors at the p100 latency. On the right, the P100 cerebral source is obtained by voltage scalp distribution and superimposed to the realistic volume conductor cortex model reconstructed from the individual anatomical magnetic resonance images. Localization was performed by means of the Curry 6.0 (Neuroscan) analysis software. For an overview on the localization procedures see Darvas et al.^39^

The EEG signal is originated by the simultaneous activation of a large number of neurons [Fig. 2(a)]. Typical values of voltage detected on the scalp are of the order of μV. The International 10–20 system is the recognized method for applying the EEG sensors on the scalp in the standard locations. The system is implemented to guarantee the reproducibility of the EEG measurements in the same subject over time and the comparability of measurements across different subjects. Usually, the EEG sensors are allocated over a cup in positions following the 10–20 system. Their number varies from 16 to 256 sensors for high-density arrays. To ensure low impedance (typical values for the more diffuse EEG amplifiers: 5 kΩ) and a good electric contact with the scalp, surface conductive gels or electrolyte-based solutions are used. Effort has been made to find solutions that allow quicker measurement preparation and a better signal-to-noise ratio. For example, dry and/or active electrodes have been tested. Dry electrodes do not need to use conductive paste whereas active electrodes amplify the EEG signals directly at the scalp, reducing the effect of ambient electrical noise.

EEG signals are a superimposition of oscillations at different frequencies and different amplitudes, with topographic and task-related temporal specificity. These oscillations are known as “brain rhythms” and can be quantified and described by means of frequency and time-frequency signal processing [Fig. 2(b)]. Brain rhythms have been traditionally classified by their frequency. Their modulations have been described and related to healthy physiological brain activity and their alteration linked to brain pathological conditions. The first rhythm observed by Hans Berger through EEG in the 20’s was an oscillation at around 10 Hz, the alpha rhythm (8 to 12 Hz), located in the occipital-parietal cortical area in eyes-closed, awake, and relaxed healthy adults. The magnitude of this rhythm was reduced after the opening of eyes or during arousal increase. For this reason, alpha has been considered related to drowsiness. Recently, it has been proposed that alpha rhythm modulation plays a role in conscious perception, as well as in the sensory gating mechanisms deployed by attention or by task-related neurocognitive strategies.^40^^,^^41^ The beta band (15 to 30 Hz) has traditionally been considered a “motor” rhythm. Indeed, beta oscillation modulations have been observed during motor and sensory processing and control, corticospinal coupling in isometric contractions, proprioception, and sensory-motor integration. The suppression of alpha and beta rhythms is the sign of the engagement of the area during the task [event-related desynchronization (ERD);^42^ Fig. 2(b)]. A recent hypothesis proposed the presence of beta activity in the maintenance of the brain “status quo” during sensory and cognitive processing.^43^ Gamma oscillations (30 to 90 Hz) have been observed in small cortical areas during several task and conditions: sensorimotor or multisensory integration, stimulus selection, feature extraction and integration, pattern recognition, pain processing, empathy, attention, and memory. A significant low-frequency activity (delta band: 1 to 4 Hz) has been observed during sleep in the healthy population. The presence of intense delta rhythm in resting state EEGs of waking adults is often associated with neurological disorders. Finally, theta band power (4 to 7.5 Hz) has been found in emotional arousal and working memory (WM) tasks, as well as in the processing and control of novelty and unexpected stimuli.

When the phase of continuous oscillations of a neuronal population is reset in response to an external stimulus or an event, an evoked response occurs. The evoked potentials (EPs) or event-related potentials (ERPs) generally are obtained by averaging time-locked to a sequence of stimuli [Fig. 2(c)]. The hypothesis is that while the responding brain area activity is identical for each stimulus, the rest of the brain activity is completely uncorrelated to it. Therefore, this uncorrelated noise that hides the activity of interest can be suppressed with averaging [Fig. 2(c)].

## 3 Combined fNIRS–EEG: Advantages and Applications

fNIRS–EEG systems can be employed with similar flexibility to stand-alone EEGs. The integration of the two methodologies provides complementary information about electrical and metabolic-hemodynamic activity of the brain cortex with no electro-optical interference. fNIRS and EEG can be combined for concurrent acquisition in a nonlaboratory environment (e.g., naturalistic environment, ambulatory monitoring, incubator, at the bedside, etc.) without causing major discomfort to the patient.^44^

**Fig. 3:**
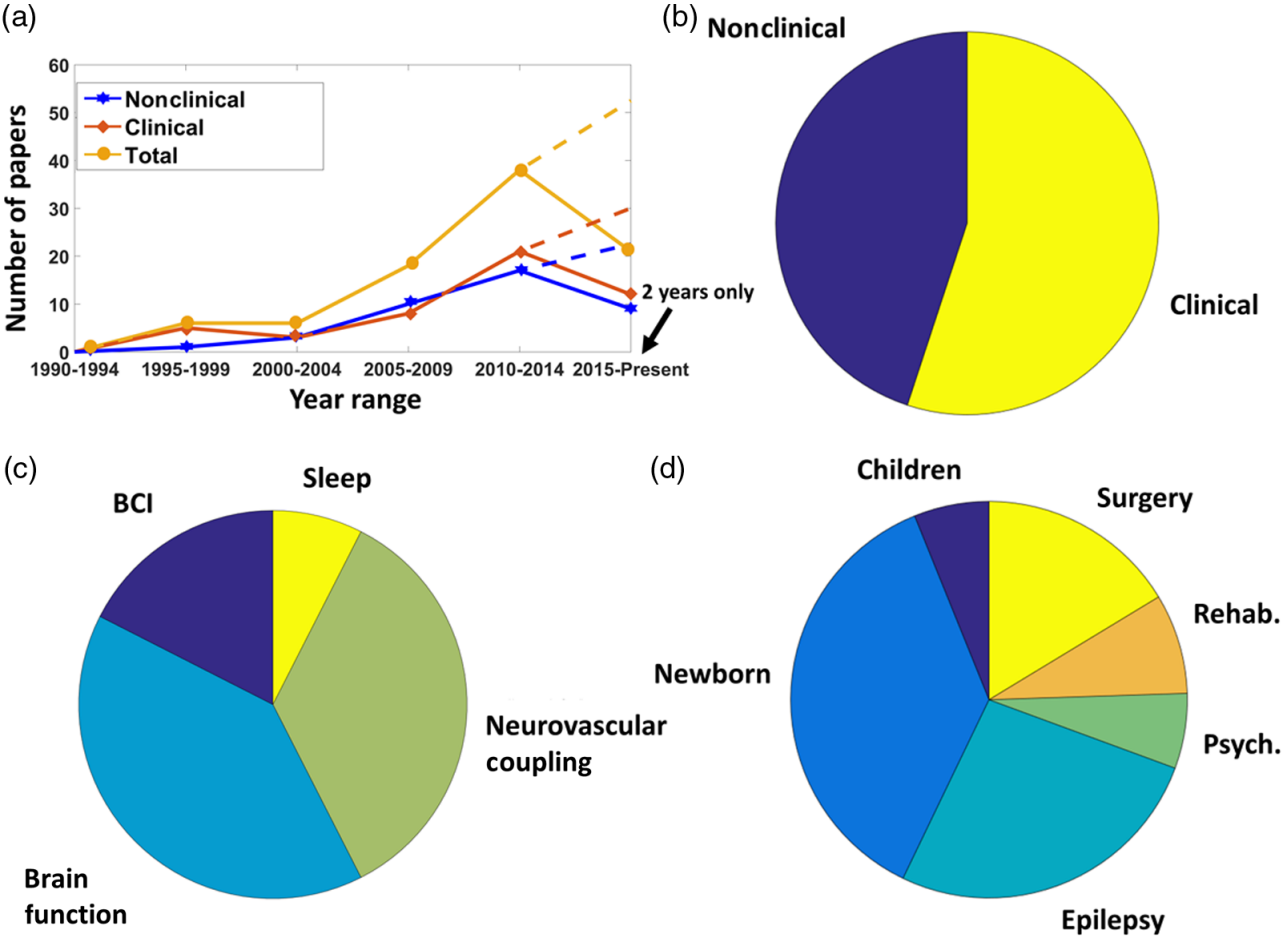
(a) Numbers of scientific papers published from 1990 to 2016 grouped at a 5-year pace. The papers are reported divided by macroapplication (nonclinical and clinical) and as a total. The dashed lines represent the expected publication at the end of 2019, since the last group of papers was produced in only 2 years (2015 to 2016). (b) Pie chart separating the papers by macroarea. (c) Pie chart separating nonclinical papers by area of interest. (d) Pie chart separating clinical papers by area of interest.

Main applications of combined fNIRS and EEG focused on both nonclinical and clinical topics. Nonclinical applications generally employed denser optical and electrical sensor arrays when compared with clinical applications. Figure 3(a) reports the total number of scientific, innovative papers published utilizing integrated fNIRS–EEG measurements, at 5 year intervals (font: Medline). Although the literature research probably does not summarize the whole scientific production, we found more than 90 scientific papers published in the last three decades using this multimodal brain imaging modality. Scientific works combining the two technologies started in the early 90s and continued to increase over time, with a small dip at the start of the new century. The dashed lines report the expected publication production at the end of 2019, since last year’s papers were counted based on a 2 year period (2015 to 2016). Comparable production over time was found for clinical and nonclinical applications, with a small prevalence of nonclinical applications before 2010 and clinical application after 2010. As reported in Fig. 3(b), similar overall scientific production came from nonclinical and clinical applications, with slightly more papers in clinical research (45% nonclinical and 55% clinical).

### 3.1 Nonclinical Applications

Within nonclinical applications, three main areas exploited fNIRS–EEG integration: brain–computer interface (18% of nonclinical applications), neurovascular coupling (35%), and the study of healthy brain functions (40%). A minor field in the nonclinical research was found to be sleep investigation (7% of nonclinical applications) [Fig. 3(c)].

#### 3.1.1 Brain–computer interface

One of the main applications of fNIRS–EEG systems is brain–computer interface (BCI).^45^^,^^46^ BCI is particularly suited for fNIRS–EEG due to the system’s flexibility and portability.

BCIs allow the control of computers or external devices directly from the modulation of brain activity due to EEG and/or other recording modalities. In fact, it was demonstrated that noninvasive EEG-based BCIs allow brain-derived communication in paralyzed and locked-in patients. Moreover, some degree of movement restoration was achieved with noninvasive BCIs in patients with spinal cord lesions and chronic stroke.^45^ Historically, the discovery of ERD and event-related synchronization (ERS) of alpha and beta rhythms in the EEG signal from motor cortex paved the way for the development of BCIs. In fact, the modulation of brain rhythms in the motor cortex during the imagination of the movement was the first parameter used for feature extraction to achieve a machine control. However, EEG-standalone BCIs still have limited capabilities. Reliable detection of BCI commands is proportional to the EEG epoch length, which makes high information transfer rates difficult to achieve. Moreover, the EEG-BCIs often misclassify the EEG signals as commands, although the subject is not performing any task.^47^ Finally, EEG signals are generally a mixture of neural activity from broad areas, some of which may not be related to the task targeted by BCI, hence impairing BCI performance. Combined fNIRS–EEG systems showed increased sensitivity and specificity when compared with a standalone EEG. The fNIRS signal can be used in a joint classification procedure with EEG or perhaps as a predictor of EEG activity. In both cases, more robust EEG-based BCI classifiers and overall an increase in the general stability of BCI performance is found using fNIRS–EEG systems.

Fazli et al., Almajidy et al., and Koo et al.^48^^–^^51^ applied combined fNIRS–EEG-BCI to sensorimotor imagery. The goal of sensorimotor imagery BCI is to identify when the subject is imagining a specific motor task. Fazli et al.^48^^,^^49^ estimated that EEG-based BCI control could be predicted by preceding fNIRS activity. The fNIRS predictions were employed to generate new, more robust, EEG-based BCI classifiers, which enhanced classification significantly while minimizing performance fluctuations and increasing the general stability of BCI. Simultaneous measurements of fNIRS and EEG on 14 subjects (24 measurement channels located on frontal motor and parietal areas for fNIRS, 37 electrodes over the whole head for EEG) improved the classification accuracy of motor imagery in over 90% of considered subjects and on average increased performance by 5%. Almajidy et al.^50^ applied BCI to four motor imagery tasks (7 subjects, 20 fNIRS channels, and 8 EEG sensors over the sensorimotor cortices): imagery motion of left hand, right hand, both hands, and both feet. The slope in $O_{2}\mathrm{Hb}$ concentration fluctuations measured by fNIRS and the power spectrum density of EEG (8 to 30 Hz) were used for feature extraction. Through linear discriminant analysis, they obtained a highest classification accuracy of 85%. Combined fNIRS–EEG-BCI showed improvement in classification accuracy when compared with EEG-BCI. Koo et al.^51^ focused on fNIRS–EEG self-paced motor imagery BCI. They performed measurements on six healthy subjects over the primary motor cortices with eight fNIRS channels and six EEG sensors. They reported that the hybrid system had a true-positive rate of about 88% and a false-positive rate of 7% with an average response time of 10 s.

Khan et al.^52^ applied a combined fNIRS–EEG system in a different fashion. They tried to extract and decode four different types of brain signals from 12 volunteers. Twelve fNIRS channels were located over the prefrontal brain region whereas eight EEG electrodes were located over the left and right motor cortex areas. The subject undergoing the BCI experiment was instructed to perform four types of tasks: “forward,” “backward,” “left,” and “right” commands. The control commands for forward and backward movements were estimated by performing arithmetic mental tasks, and they were related to $O_{2}\mathrm{Hb}$ changes. The left and right directions commands were associated with right and left hand tapping, respectively. High classification accuracies were achieved for the four different control signals using the hybrid fNIRS–EEG technology.

EEG-BCIs can be also performed through steady-state visually evoked potentials (SSVEP) classification. SSVEP are signals that are natural responses to visual stimulation at specific frequencies. When the retina is excited by a visual stimulus ranging from 4 to 80 Hz, the brain generates electrical activity at the same frequency or at multiples of it. The goal is to discriminate when the subject is looking at the stimulus and to detect the frequency of the stimulus with high accuracy. Tomita et al.^53^ focused on the joint use of fNIRS and EEG during an SSVEP classification on 13 subjects. The authors showed a clear improvement in error rates obtained by combining only one fNIRS channel with EEG measurements.

Finally, fNIRS-based prior information was incorporated in a variational Bayesian multimodal encephalography methodology.^54^ The authors applied a Bayesian logistic regression technique to decode subjects’ mental states to a spatial attention task in a unified fNIRS–EEG framework. The authors found that the fNIRS–ERD-based decoder exhibited significant performance improvement over decoding methods based on EEG sensor signals alone (8 volunteers, 49 fNIRS channels over the parietal and occipital lobes, and 64 EEG electrodes).

#### 3.1.2 Neurovascular coupling

Localized neural activity is accompanied by complex and heterogeneous biological processes, such as electrical activity generation and concurrent metabolic variation. The underlying link between electrical events and hemodynamic oscillations caused by the metabolic activity is generally referred to as neurovascular coupling.^55^ Through vasodilatory processes an overcompensatory oxygenated blood supply is provided to activated brain areas. Combined fNIRS–EEG measurements are highly suited for neurovascular coupling monitoring both in a data-driven approach or, if the coupling is assumed known, for better neural activity reconstruction. An important aspect of neurovascular coupling is that electrical brain activity and its hemodynamic response do not have a perfect spectral and spatiotemporal correspondence, nor, although often it is assumed for simplicity, is their relation linear. Neurovascular coupling can be assessed in different experimental settings: during resting state activity, in response to an external sensory stimulation, and during manipulation of vascular parameters and manipulation of brain electrical states.

Several authors^56^^–^^59^ studied neurovascular coupling through fNIRS–EEG measurements on the brain at rest. Keles et al.^56^ collected data with a combined sparse whole-head fNIRS–EEG system (18 subjects, 19 recording locations, combined fNIRS–EEG sensors). Focusing on spectral-EEG effects on neurovascular coupling, they found a delayed alpha activity (8 to 16 Hz) modulation in occipital areas and a strong beta activity (16 to 32 Hz) modulation of hemodynamic fluctuations, which was generated by the alpha-beta coupling in EEG. Nikulin et al.^57^ also focused on the link between specific spectral EEG features and neurovascular coupling at rest. They studied the monochromatic ultraslow oscillations (0.07 to 0.14 Hz) in human EEG and their relation with hemodynamic (10 subjects, 26 fNIRS channels, and 58 EEG electrodes). They suggested that these oscillations might be of a rather extraneuronal origin reflecting cerebral vasomotion. Pfurtscheller et al.^58^ performed motor cortices EEG and frontal fNIRS measurements on nine volunteers and found that slow precentral HHb concentration oscillations during awake rest could be temporarily coupled with EEG fluctuations in sensorimotor areas. Attempts for continuous quantification of neurovascular coupling in spontaneous brain activity have been made by Govindan et al.^59^ The author proposed a method for neurovascular coupling quantification based on combined fNIRS–EEG acquisitions. They brought the two measurements into a common dynamical time frame (DTF), by partitioning both signals into 1-s epochs. They quantified the extent of neurovascular coupling by calculating spectral coherence between the two signals in the DTF. The authors tested their procedure on both simulated data and four infants undergoing therapeutic hypothermia for neonatal encephalopathy with encouraging results. In particular, they found increased neurovascular coupling based on their metric in children who showed the best recovering.

Neurovascular coupling can also be assessed during time-locked brain responses to external stimulation.^8^^,^^60^ Obrig et al.^60^ combined fNIRS and EEG during visual stimulation (visual evoked potential for EEG, VEP) in the assessment of both electrical and hemodynamic visual habituation on 15 volunteers (two fNIRS channels over the occipital cortex and five EEG sensors). Within the stimulation period, they found a decrease in P100/N135-component amplitude, closely coupled to a decrease in the amplitude of tissue oxygenation. Unclear neurovascular behavior was found when considering the N75/P100-component. Although stressing the approximation implied assuming neurovascular coupling a linear phenomenon, they found, when calculating a ratio between the amplitude of the P100/N135-component and the concentration changes in the hemoglobin, a coupling index of 0.2  μmol in HHb and of +0.6  μmol in $O_{2}\mathrm{Hb}$ per 1  μV increase in VEP-component amplitude. Also, Fabiani et al.^8^ investigated the relationship between neuronal and hemodynamic changes elicited by visual stimulation by combining fNIRS–EEG measurements over the occipital cortex (32 source and 8 detector locations for fNIRS and 7 EEG sensors), together with fast optical signals^61^^,^^62^ and functional MRI. Based on the C1 location response of the ERP for the electrical components, results indicated that both younger (19 subjects) and older adults (44 subjects) exhibited a nonlinear (at least quadratic) relationship between neuronal and hemodynamic effects, with reduced the hemodynamic response at high levels of neuronal activity.

Neurovascular coupling relying on vascular state manipulation was further investigated.^63^^,^^64^ Babiloni et al.^63^ investigated vasomotor reactivity and coherence of resting state EEG rhythms in the older population during hypercapnia. Resting state eyes-closed fNIRS–EEG data were recorded pre-, during-, and posthypercapnia in 20 subjects. Frontal bilateral fNIRS (two channels) was performed to assess the vasomotor reactivity estimate through cortical HHb and $O_{2}\mathrm{Hb}$ concentration changes. EEG coherence across all electrodes (19 sensors) was computed at different EEG frequency bands. Hypercapnia increased $O_{2}\mathrm{Hb}$ and decreased HHb as well as total EEG coherence. Moreover, they found that the extent of changes in these variables and hypercapnia were strictly correlated, reflecting in a neurovascular coupling phenomenon. Vanhatalo et al.^64^ applied combined fNIRS–EEG recordings on 12 subjects to study whether hemodynamic changes in human brain generate slow frequency EEG responses. They employed 24 fNIRS channels over the frontal-parietal regions and six EEG channels. They applied noninvasive manipulations of intracranial hemodynamics by different mechanisms: bilateral jugular vein compression, head-up tilt, head-down tilt, Valsalva maneuver, and Mueller maneuver. They observed consistent slow EEG shifts during all manipulations with highest amplitudes (up to 250  μV) at the midline electrodes, and the most pronounced changes (up to 15  μV/cm) in the voltage gradient around the vertex. Their interpretation of extraneuronal origin of slow EEG oscillations was analogous to the one found later by Nikulin et al.^57^

Assessment of neurovascular coupling through fNIRS–EEG during anodal transcranial direct current stimulation (tDCS) of brain electrical activity was performed by Dutta et al.^65^^–^^67^ Based on analysis procedures relying on the Hilbert–Huang transform, they analyzed chronic stroke survivors and found nonstationary effects of anodal tDCS on EEG that correlated with the $O_{2}\mathrm{Hb}$ response. They also found correspondence between the initial dip in $O_{2}\mathrm{Hb}$ concentration at the beginning of anodal tDCS and an increase in the mean-power of EEG within a 0.5- to 11.25-Hz frequency band.

Finally, approaches to the neurovascular coupling relying on mathematical models of the phenomenon have been proposed. A mixed model-based data-driven approach was provided by Talukdar et al.^68^ They applied gamma transfer functions to map EEG spectral envelopes that reflect time-varying power variations in neural rhythms to hemodynamics measured during median nerve stimulation. They first validated the approach using simulated fNIRS–EEG data and then applied the procedure to experimental fNIRS–EEG recordings (16 sources and 8 detectors for fNIRS, 30 EEG electrodes, whole head). By applying cluster analysis, statistically significant parameter sets were found to predict fNIRS hemodynamics from EEG spectral envelopes. The three subjects investigated were found to have significant clustered parameters for fNIRS–EEG data fitted using gamma transfer functions. Results from the experimental data indicated that the neurovascular coupling relationship could be modeled using multiple sets of gamma transfer functions and the approach could provide a better understanding of neurovascular coupling phenomenon.

#### 3.1.3 Brain Functions

fNIRS–EEG has been involved in the characterization of the healthy brain functions. Within this field of application, fNIRS–EEG allowed investigation in an ecological environment of the spatiotemporal hemodynamic and electrical evolution of brain activity during sensory stimulation, language, motor intention, WM, and emotions in social interaction or stressful event. Part of the combined fNIRS–EEG studies focused on characterizing brain activity during external sensory stimulation (auditory or visual).^69^^–^^71^

Ehlis et al.^69^ conducted simultaneous fNIRS–EEG measurements (22 fNIRS channels on frontotemporal areas and 3 midline EEG electrodes) on 10 subjects to assess cortical correlates of auditory sensory gating in humans. Sensory gating refers to the ability of cerebral networks to inhibit brain response to irrelevant environmental stimuli to prevent information overflow. Acoustic gating is generally assessed based on the reduction of the P50 amplitude (an early component of the ERP in electrophysiological recordings) after repeated occurrence of a particular acoustic stimulus. Combining the hemodynamic data with electrophysiological information revealed a positive correlation between the amount of sensory gating and the strength of the hemodynamic response in the left prefrontal and temporal cortices. The results strengthen the hypothesis of a possible inhibitory influence of the prefrontal cortex on primary auditory ones. Takeda et al.^70^ recently studied the effect of pleasant and unpleasant auditory stimulation on the prefrontal cortex of 12 subjects with combined fNIRS–EEG measurements and other autonomic nervous system monitoring. The authors found a bilateral increase in the hemodynamic activity of the prefrontal cortex with a greater activation of the left side for pleasant stimuli and a larger activation of right side for unpleasant stimuli. Greater alpha wave modulation was obtained for pleasant versus unpleasant stimulation.

Regarding sensory-related brain response investigation, visual stimulation has been studied using combined measurements. Rovati et al.^71^ utilized an embedded few channels fNIRS–EEG instrumentation for the assessment of hemodynamic changes and VEP in the occipital area of nine subjects during steady-state visual stimulation, employing different stimulus contrasts (1%, 10%, and 100%). The results showed clear, coupled, BOLD, and VEP responses. Both responses, hemodynamic and VEP, presented a logarithmic profile as a function of the stimulus contrast.

Jaušovec and Jaušovec^72^^,^^73^ exploited gender differences in the brain processing during visual and auditory stimulation using fNIRS–EEG (eight fNIRS channels located over left-right frontal cortex in a multidistance configuration and 19 EEG sensors) measurements on 30 males and 30 females. The fNIRS results showed that males have a higher increase in oxygen saturation during task performance compared with females. Gender-related differences in EEG activity were observed in the amplitudes of the early evoked gamma response and the P3 component and were more pronounced for the visual than for the auditory stimuli. Overall, the studies suggested that females’ visual event-categorization process is more efficient than in males.

Other multimodal experiments focused on sensorimotor responses and motor intention paradigm.^74^^,^^75^ Takeuchi et al.^74^ investigated hemodynamic responses and neural activity relationships in the somatosensory cortices of 18 young adults during electrical stimulation of the right median nerve. They developed a head cap for fNIRS and EEG in a square grid configuration employing whole-head 103 fNIRS and 32 EEG channels. A GLM-based analysis of the hemodynamic signal showed increased $O_{2}\mathrm{Hb}$ concentration at the contralateral primary somatosensory region during stimulation, followed by responses that spread to more posterior and ipsilateral somatosensory areas. The EEG data indicated that positive somatosensory evoked potentials (SEPs) peaking at 22-ms latency (P22) were recorded from the contralateral somatosensory area. fNIRS and EEG topographical maps of hemodynamic responses and current source density of P22 were significantly correlated. Furthermore, time-delayed GLM-analysis highlighted the temporal ordering of neural activation in the hierarchical somatosensory pathway. Pfurtscheller et al.^75^ investigated whether the initiation of a voluntary motor act, such as finger movement, could be temporally related to slow $O_{2}\mathrm{Hb}$ and electrical oscillations at rest. They analyzed, in a continuous fashion, prefrontal HHb and $O_{2}\mathrm{Hb}$ with fNIRS and EEG signals (few channels systems) over sensorimotor and prefrontal areas in 10 healthy subjects at rest. They found that EEG beta power increases ∼3  s after slow fluctuating $O_{2}\mathrm{Hb}$ peaks during rest was indicative of a slow excitability change of central motor cortex neurons that could possibly trigger the voluntary motor act.

Some studies focused their attention on the proprioception-related brain state during changes of gravity conditions. The influence of changing gravity conditions on neurophysiological processes and associated neurocognitive impairment is of critical interest for aerospace application.^76^^,^^77^ Brümmer et al.^76^ highlighted the suited characteristic of fNIRS–EEG imaging for assessing neurophysiological processes during atypical gravity conditions in two subjects. Smith et al.^77^ investigated the relationship between brain cortical activity (through 32 EEG sensors) and brain oxygenation (through a two channel fNIRS system) in the prefrontal cortex of 12 participants during hypergravity exposure. In fact, artificial gravity has been proposed as a method to counteract the physiological deconditioning of long-duration spaceflight; however, the effects of hypergravity on the central nervous system were poorly assessed. The authors found a significant increase in the EEG prefrontal cortex activity during hypergravity. Moreover, prefrontal cortex oxygenation was significantly decreased during and because of hypergravity exposure. However, no significant correlation was found between EEG prefrontal cortex activity and hemodynamic variables. Thus, the authors concluded that the increase in the EEG prefrontal cortex activity could be attributable to psychological stress, which could pose a problem for the use of a short-arm human centrifuge as a countermeasure.

Some work was also conducted on studying neural correlates of attention and WM.^78^^,^^79^ Butti et al.^78^ investigated and described neural correlates during sustained attention in nine subjects through the usage of a 16-channel fNIRS and 19-channel EEG system. Good agreement was found between the two modalities, both showing higher brain activity in the middle upper frontal and temporal regions during the task. Jaušovec and Jaušovec^79^ investigated the effect of training on WM tasks in 30 participants through changes in fNIRS–EEG (8 and 19 channels, respectively) patterns of brain activity. WM training significantly increased performance on all tests of fluid intelligence. During WM, changes in patterns of EEG brain activity were mostly pronounced in the theta and alpha bands for the trained group. Theta and lower-1 alpha band ERS was accompanied by increased lower-2 and upper alpha ERD. The hemodynamic patterns of brain activity after training changed from higher right hemispheric activation to a balanced activity of both frontal areas. The electrical as well as hemodynamic patterns of brain activity suggested that the training influenced WM.

Moreover, fNIRS–EEG systems were applied to study language-related processes. Language involves auditory and visual task as well as more complex brain mechanisms. Thus, a multimodal brain imaging approach is almost a requirement for its characterization.^80^ fNIRS–EEG coregistration combines high temporal and spatial resolution and can offer unique opportunities for studying functional connectivity in linguistic experiments. Measurement of electrical and metabolic brain activities is essential, and the multimodal approach should be noninvasive to facilitate *in vivo* recordings, particularly in children. Wallois et al.^80^ reviewed the advantages of simultaneous fNIRS–EEG acquisition in providing a better understanding of the brain mechanisms involved during language processes.

Emotion perception is also a complex process that should preferably be examined by means of a multimethodological approach. fNIRS presents several advantages in the study of emotions when compared with other approaches, especially in combination with frequency resolved EEG.^81^ Among the different modalities available for monitoring brain activity, fNIRS is particularly well suited for evaluating the prefrontal cortex activity, which is among the regions involved in emotional processing. Balconi et al.^81^ investigated the brain processing of emotional images with fNIRS–EEG 6-channel fNIRS system over the prefrontal area and 16-channel EEG system). The 20 subjects undergoing the study were asked to observe and evaluate affective pictures. The multiple measures were then related to self-report data such as subjective appraisal in term of valence (positive versus negative) and arousal (high versus low). The contribution of prefrontal cortex was elucidated by the $O_{2}\mathrm{Hb}$ increase within the right hemisphere with negative valence, suggesting a relevant lateralization effect induced by the specific valence (negative) of the emotional patterns. Moreover, EEG activity, especially in theta and delta bands, was associated with the cortical hemodynamic responsiveness to the negative emotional patterns within the right side. Important effects were derived from correlational analyses between hemodynamic and cortical EEG. Other studies evaluating the response to pleasant and unpleasant emotions were conducted by Hoshi et al.^82^ Nineteen subjects were exposed to negative, positive, and neutral pictures previously classified. fNIRS (through 16 channels located on the forehead), ERPs (through six EEG electrodes), systemic blood pressure, and pulse rate were measured simultaneously. Unpleasant emotion was accompanied by an $O_{2}\mathrm{Hb}$ increase in the bilateral ventrolateral prefrontal cortices, while very pleasant emotion was accompanied by a decrease in $O_{2}\mathrm{Hb}$ in the left dorsolateral prefrontal cortices. Brain activation of the occipital cortex modulated by the emotional content of the visual positive and negative stimuli has been studied by Herrmann et al.^83^ with combined fNIRS–EEG (22 and 4 channels, respectively) on 16 volunteers. The ERP results showed an increased early posterior negativity over the occipital cortex for both positive and negative stimuli. Moreover, positive as well as negative stimuli lead to a significantly higher decrease in HHb than neutral stimuli over the occipital cortex. This result can be explained by the selective attention occurring while viewing pictures with an emotional content. The use of fNIRS as an alternative technique for mental state analysis has been also studied^84^ and compared with other conventional techniques such as EEG and peripheral arterial tonometry. Seven subjects were exposed to stress and healing tasks, and a 6-channel fNIRS and 10-channel EEG signals were recorded over the frontal area. fNIRS results showed increased HbT in the frontal cortex during the stress task and decreased HbT during the healing phase for all subjects that were sensitive to the specific stimuli.

fNIRS–EEG recordings are also highly suited for studying social interaction.^85^^,^^86^ The relevant approaches to studying brain state during social interaction were reviewed in Konvalinka and Roepstorff.^85^ Reviewed studies employed either fMRI, EEG, or fNIRS and the recordings coupling each other in quantifying hemodynamics or modulations of brain rhythms, both intra- and interpersonally, and integrate various conceptual frameworks. Regarding social interaction, fNIRS and EEG were employed in a hyperscanning modality^86^ where multiple subjects are recorded using the same instrument. Hyperscanning approaches were already described for fMRI dual recording coil. This approach is highly suited for studying social interaction since there is no need to calibrate across devices, there are no synchronization problems, and experimental designs are easy to implement.

#### 3.1.4 Sleep

Multimodal fNIRS–EEG recordings can be suited for long brain monitoring during sleep due to the potential flexibility and portability of the combined technology. Combined fNIRS–EEG measurements were applied to the study of sleep phases or awake to sleep transitions. In this context, EEG mainly provided information about different sleep conditions (such as non-REM phase, REM phase, or awake-to-sleep transition), whereas fNIRS estimated the hemodynamic fluctuations of hemoglobin during the different phases. Pierro et al.^87^ investigated amplitude and phase of spontaneous low-frequency oscillations (LFOs) of cerebral HHb and $O_{2}\mathrm{Hb}$ concentrations during sleep in five subjects employing two multidistance probes located on the forehead. Interestingly, by applying phasor algebra, they were able to estimate oscillations in CBV and cerebral blood flow velocity. By exploiting phase differences between the two forms of hemoglobin, they found greater phase lead of HHb versus $O_{2}\mathrm{Hb}$ LFOs during non-REM sleep with respect to the awake and REM sleep states (∼π/2). Amplitude analysis highlighted suppression of both forms of hemoglobin during non-REM sleep with respect to the awake and REM sleep states (maximum amplitude decrease: 87%). The associated CBV and CBFC oscillations were found to maintain their relative phase difference during sleep, and their amplitudes were attenuated during non-REM sleep. Overall, the author highlighted the capabilities of phasor algebra to the study of LFO during sleep phases. Some work to characterize intracerebral hemodynamic during sleep state transitions has also been performed.^88^ By studying frontal areas (employing one fNIRS and seven EEG channels) in nine subjects, a decrease in HbT concentration at sleep onset and an increase in HbT concentration at sleep offset were highlighted with an average effect lasting ∼3  s. The results suggested an overcompensatory increase in brain perfusion during wakefulness with respect to sleep.

Pizza et al.^89^ were the only ones that investigated fNIRS–EEG temporal relation during sleep. In particular, they estimated the synchronized signal changes associated with periodic leg movements during sleep (PLMS) in three subjects employing eight fNIRS channels and four EEG channels. PLMS is a movement disorder that occurs during sleep sometimes called periodic limb movements during sleep. They found that PLMS were constantly associated with increased oscillations amplitude in cerebral hemodynamic, and they were coupled with changes in the EEG features.

### 3.2 Clinical Applications

Clinical research mainly focuses on newborn (37% of clinical applications) and epilepsy (27%) [Fig. 3(d)]. Although slightly less applied, fNIRS–EEG found good application in the intraoperative environment (surgery, 16%). Minor clinical applications were rehabilitation (8% of clinical applications), child development (6%), and psychiatry (6%).

#### 3.2.1 Newborn

The continuous monitoring of neurological functioning is mandatory in critically ill preterm and full-term infants. Due to its noninvasiveness and portability, EEG is the technique most suited to continuously monitor electrical brain activity. Indeed, the use of the amplitude-integrated electroencephalography (aEEG) is routinely employed in neonatal intensive care units: a limited number of EEG channels, typically a pair over bilateral parietal or central regions, are placed on the neonate scalp and data are displayed in a semilogarithmic time-compressed scale. The usefulness of EEG has been proven in premature infants, full-term infants with hypoxia-ischemia, and infants suspected of epileptic seizures. In this scenario, fNIRS is utilized to noninvasively collect parameters that characterize metabolic activity, generally employing few optodes. Changes in HHb and $O_{2}\mathrm{Hb}$, ${\mathrm{rSO}}_{2}$, and FTOE provide a way to continuously monitor brain oxygen imbalance. Regarding NIR investigation of newborns’ brain status, it is worth highlighting that, although not strictly related to the topic of this review, combined FD-NIRS and diffuse correlation spectroscopy (DCS)^90^^–^^92^ measurements are arousing great interest within the fNIRS and neonatal communities. In fact, FDNIRS-DCS systems allow for quantitative assessment of cerebral blood flow in preterm infants^93^ and infants with hypoxic encephalopathies^94^^,^^95^ or congenital cardiopathies.^96^^,^^97^

The integration of fNIRS and EEG techniques will probably become the future direction of neonatal brain monitoring.^98^^,^^99^ Nowadays, the simultaneous measures are usually performed synchronizing two different conventional fNIRS and EEG systems. In particular, few fNIRS sensors are applied over the bilateral parietal or temporal regions together with the EEG.

A low oxygen delivery may be the cause of brain damage in preterm and term newborns. Nowadays, brain cooling is the elective treatment in asphyxiated newborns, and combined fNIRS and EEG may be a valuable method to monitor CBV, brain oxygenation, and electrical activity during hypothermia, possibly revealing its efficacy.^100^ Moreover, several studies have been carried out to investigate the prognostic value of fNIRS–EEG brain monitoring in asphyxia, without conclusive findings.^101^^–^^105^ The efficacy of fNIRS in monitoring cerebral oxygenation to guide treatments within the first 72 h of life has been also studied in clinical trials: in fNIRS-guided-treated infants, a reduction in the burden of cerebral hypoxia, as evidenced by EEG outcomes, has been shown.^106^^,^^107^

Combined NIRS and EEG have been utilized to characterize brain functioning alterations in infants. In patients with neurological damage, in which seizures were observed, transient hemodynamic events frequently occurred.^108^ In preterm infants, the combined techniques may give new insights into brain functioning, to identify potentially vulnerable conditions after birth and to better understand the required treatments.^109^ Indeed, increased maturation of EEG activity is associated with decreased variability in cerebral oxygen extraction and was accompanied by increased FTOE. Moreover, oxygenation in the first hour after birth may be a biomarker of brain vulnerability.^110^^–^^112^

During immediate postnatal transition, i.e., within the first 15 min after birth, useful information provided by fNIRS and EEG may help to guide resuscitation.^113^ Neonates with initially low cerebral activity (i.e., low EEG activity) during immediate transition after birth concurrently showed low ${\mathrm{rSO}}_{2}$ values,^114^ and compromised neonates that require resuscitation presented different cerebral activity with respect to uncompromised neonates.^115^ Moreover, the fNIRS–EEG simultaneous brain monitoring has been utilized in monitoring brain functioning in deep hypothermic circulatory arrest during arterial switch operation.^116^^,^^117^

An important question in intensive care unit is understanding how infants respond to painful treatments that they receive.^118^ A multimodal approach for measuring brain responses to peripheral noxious and sensory stimulations in infants has been tested. The simultaneous detection of (1) brain responses through fNIRS and EEG, (2) withdrawal reflex activity through electromyography, (3) autonomic responses by means of pulse oximetry, electrocardiography, and respiration, and (4) behavioral activity through video monitoring had 100% sensitivity and specificity in measuring both types of stimulation.^119^ Note that single-trial analysis of responses to somatosensory and noxious stimuli showed that at individual levels, electrophysiologic and hemodynamic responses do not always occur together.^120^ This result highlights the need for integrated brain monitoring in newborns. As for sensory stimuli, a combined approach can also be successfully applied to investigate the newborn cerebral responses to stimuli obtained with other modalities, for example, visual responses during photic stimulation,^121^ as well as to monitor the brain ability of sensory processing during normal development, such as acoustic processing in infants within the first 6 months of life.^122^

#### 3.2.2 Children

fNIRS–EEG recordings were also applied for monitoring the brain status of ill or injured children (preschool and primary school age). fNIRS–EEG monitoring is particularly suited for studying brain response in children without causing major restraint and discomfort. This is particularly important in children with neurodevelopmental problems.

Zennifa et al.^123^ monitored the unrestrained cognitive state of children with mental retardation using wireless combined fNIRS–EEG recordings. Marx et al.^124^ investigated fNIRS for the treatment of children with attention-deficit-hyperactivity disorder (ADHD) in a neurofeedback approach. In this pilot study, $O_{2}\mathrm{Hb}$ in the prefrontal cortex of children with ADHD was measured and fed back. fNIRS-neurofeedback was compared with the EEG (slow cortical potentials) and feedback from electromyographic (EMG) signals (i.e., muscular activity of left and right musculus supraspinatus). The task was used either to increase or decrease hemodynamic activity in the prefrontal cortex (fNIRS), to produce positive or negative shifts of SCP (EEG), or to increase or decrease muscular activity. The author reported that ADHD symptoms decreased significantly 4 weeks and 6 months after the fNIRS and EEG or EMG training according to different metrics.

fNIRS–EEG measurements were also applied for monitoring of traumatic brain injury (TBI) in children.^125^ In fact, children commonly develop secondary diffuse cerebral swelling after TBI. Adelson et al.^125^ used fNIRS on children with severe TBI and compared Hb, $O_{2}\mathrm{Hb}$, and HbT fluctuations with intracranial pressure (ICP), mean arterial pressure (MAP), and EEG metrics. The researchers found increased HbT with worsening ICP and MAP parameters, indicating increased cerebrovascular dilatation after the injury. They also noticed that posttraumatic seizures were preceded by an unexplained rapid cerebral hyperoxygenation several hours prior to the onset of clinical seizures. Researchers concluded that fNIRS could be integrated with EEG and other methodologies for monitoring posttraumatic brain injuries in children.

#### 3.2.3 Epilepsy

fNIRS and EEG are feasible for long continuous monitoring as they do not require subject immobilization. This characteristic is of crucial importance in the study of patients with epilepsy; fNIRS–EEG-integrated synchronous measurements were performed extensively on epileptic patients.^126^^,^^127^

Notice that the studies presented in this section are reported by subdividing them between studies that were focused on the temporal characteristic of the signals recorded (generally employing fewer fNIRS–EEG sensors) and studies that were more interested in the signals spatial information content (generally employing more fNIRS–EEG sensors). The division was not performed based on clinical aspects such as the presence of interictal discharges, focal seizures, and/or generalized seizures.

Steinhoff et al.^128^ performed a pilot study where they coupled fNIRS recordings with video-EEG during the presurgical evaluation of two patients with intractable epilepsy of mesial temporal origin. Two fNIRS sensors were placed on the frontal cortex. Ipsilateral measurements revealed a marked desaturation during the seizures with a postictal maximum. The favorable outcome of selective amygdalahippocampectomy retrospectively confirmed the correct lateralization based on video-EEG and the fNIRS findings in both patients.

Combined studies have been performed during the last two decades mainly with the goal to assess the usefulness of fNIRS in epileptic patients and primarily focused on the hemodynamic mechanisms before, during, and after seizures (periictal phase) at different time scales and brain locations.^14^^,^^15^^,^^129^^–^^132^ For a good review regarding focal seizures and interictal epileptiform discharges identification using fNIRS–EEG recordings, refer Ref. 133.

Adelson et al.^129^ combined fNIRS–EEG to study cerebral oxygenation in the periictal phase. Ictal events were recorded and oxygen availability was evaluated in pre-, intra-, and postictal periods. Although the study was preliminary, only two patients were examined (with large age variability), and only a few optodes were employed, they found an increase in cerebral oxygenation between 1 and 10 h before the ictal event. Continued seizure activity and isolated ictal events were associated with decreased cerebral oxygen availability. Differences in cerebral oxygen availability were noted among different types of seizures. Seyal^130^ investigated whether hemodynamic changes in the frontal scalp could predict temporal lobe seizures by recording simultaneously fNIRS and video-EEG. An fNIRS sensor was placed ipsilateral to the first recorded seizure on six patients. ${\mathrm{rSO}}_{2}$ increased during the preictal phase, around 5 min prior to the seizure, and it decreased close to the seizure onset. After the seizure, ${\mathrm{rSO}}_{2}$ increased again with an overoxygenated state lasting for around 35 min. Sokoloff et al.^131^ studied 20 critically ill neonates in the periictal phase. They estimated cerebral and systemic ${\mathrm{rSO}}_{2}$ with concurrent fNIRS, over the parietal region, and video-EEG. ${\mathrm{rSO}}_{2}$ declined during seizures compared with baseline and postictal phases (baseline 81.2 versus ictal 77.7 versus postictal 79.4). FTOE was highest during seizures. Moreover, they evaluated the impact of phenobarbital administration in the infants. After drug administration, cerebral ${\mathrm{rSO}}_{2}$ rose and FTOE declined, with monotonic relations as a function of phenobarbital dosage. Roche-Labarbe et al.^14^ studied metabolic/hemodynamic brain activity during absence epilepsy in children. They measured HHb, $O_{2}\mathrm{Hb}$, and HbT with an fNIRS–EEG system (1 fNIRS channel over the left frontal area and 11 electrodes positioned according to the 10-20 system). They recorded frontal hemodynamic fluctuations in six patients with generalized spike-and-wave discharges (GSWD). GSWD were associated with an increase in tissue oxygenation in the frontal area (beginning 10 s before the GSWD) followed by a strong deoxygenation phase, another increase in oxygenation and CBV, and a final return to baseline. Regional CBV during seizures has been studied by Watanabe et al.^15^ in 12 patients with intractable epilepsy. Eight or twenty-four channel fNIRS were employed for nine and three subjects, respectively. Seizures were induced by bemegride injection. In all cases, rCBV increased rapidly after the seizure onset. The increased rCBV lasted between 30 and 60 s. Shichiri et al.^132^ monitored CBV in two patients with symptomatic epilepsy for which epileptic discharges were not recognized in the EEG. fNIRS monitoring demonstrated an increase in CBV in the right frontal region, which began 10 min before the seizure onset and lasted for 3 h.

Moreover, more sophisticated analysis was performed to improve the detection of epileptic activity using fNIRS. Machado et al.^134^ compared TD and time-frequency domain (wavelet) methods based on the GLM approach to detect hemodynamic responses during epileptic activity. The time of epileptic discharges was detected based on the EEG. This analysis was tested using both realistic simulations with different signal-to-noise ratios and on an epileptic patient. For fNIRS, the wavelet analysis was more specific than the TD analysis. Forty-three fNIRS channels (21 sources and 8 detectors) were placed over the right frontal, bilateral parasagittal regions, and bilateral rolandic regions. EEG was carried out simultaneously using 19 electrodes placed according to the 10–20 system. A focal increase in CBV was found in the 10-year-old epileptic patient in accordance with the epileptogenic focus that was confirmed after surgery. Pouliot et al.^135^ investigated posterior epilepsies employing combined high-density fNIRS–EEG recordings (over 100 fNIRS channels and 19 EEG sensors). Spikes and seizures were marked on EEG traces and convolved with a standard hemodynamic response function for GLM analysis. GLM results for seizures (in three patients) and spikes (seven patients) were broadly sensitive to the epileptic focus in seven of the nine patients examined and specific in five patients. The Hbb responses were localized in regions within the occipital or parietal lobes. The same group also reported fNIRS–EEG recordings and analysis applied to monitoring of temporal^136^ and frontal^137^ lobe seizures. The authors found a bilateral increase when the temporal seizures occurred for CBV, $O_{2}\mathrm{Hb}$, and decreased Hbb, followed by an increased Hbb. Moreover, they found heterogeneous hemodynamic changes in remote frontal and/or parietal areas early on when epileptic activity was limited to the temporal lobe. Hemodynamic changes in the frontal lobe seizures consisted of lateralized and local increases of CBV and $O_{2}\mathrm{Hb}$ but heterogeneous Hbb behavior. Furthermore, rapid hemodynamic alterations were observed in the homologous contralateral region, even in the absence of obvious propagated epileptic activity.

Other studies involving fNIRS–EEG measurements were directed toward epileptogenic focus localization.^138^^–^^140^ Watanabe et al.^138^ examined the use of multichannel fNIRS (24 channels) to evaluate CBV change during long-term EEG monitoring in 32 cases of intractable epilepsy. The goal was to locate the epileptogenic focus. In 96% of cases, fNIRS showed significant hyperperfusion in the side of seizure foci, whereas ictal single-photon emission computed tomography SPECT showed hyperperfusion in 69% of cases. Peng et al.^139^ performed a simultaneous fNIRS–EEG study using a large bilateral coverage (64 fibered light sources and up to 16 detectors, 19 carbon EEG electrodes) on 40 patients with drug-resistant focal epilepsy. They generated topographic maps of hemoglobin fluctuations caused by interictal epileptic discharges. They reported a significant variation of HHb in 62% of patients with neocortical epilepsies where the epileptic foci were identified. Presurgical investigation of patients with refractory epilepsy has been also studied. Gallagher et al.^140^ recorded fNIRS–EEG signals to evaluate the position of the ictal onset zone (28 fNIRS channels over the right frontal bilateral parasagittal regions and bilateral rolandic regions, 18 EEG electrodes). One patient underwent a prolonged recording, and the results were compared with other presurgical techniques. They obtained a good concordance on the ictal onset zone localization showing that combined fNIRS–EEG could be used to increase presurgical investigation accuracy. In a recent study,^141^ the same group evaluated the possible presurgical usefulness of fNIRS–EEG in a boy with refractory epilepsy. In the study, they compared high-density fNIRS (11 detectors and 46 sources) results obtained while the participant performed expressive and receptive language tasks with those obtained using fMRI. The case study illustrated the potential for fNIRS to contribute favorably to the localization of language functions in children with epilepsy and cognitive or behavioral problems and its potential advantages over fMRI in a presurgical assessment.

The usefulness of fNIRS in the medication management of an infant with status epilepticus and subtle or no clinical manifestations was studied by Arca Diaz et al.^142^ Simultaneously, monitoring of electroencephalographic activity and cerebral ${\mathrm{rSO}}_{2}$ was performed. They found that antiepileptic drugs influenced the frequency of ${\mathrm{rSO}}_{2}$ fluctuations and electroencephalographic seizures. They suggested that fNIRS could be used to gauge the effects of antiepileptic medications in patients with similar disease manifestation. In another recent study, Visani et al.^143^ evaluated the hemodynamic and EEG signals during unilateral hand movement in patients with cortical myoclonus. TD-fNIRS–EEG (16 fNIRS channels over the sensorimotor areas, centered over C3 and C4, and 19 EEG sensors) together with fMRI data were acquired. Ten patients with progressive myoclonic epilepsy and 12 healthy controls underwent the measurements. HHb, $O_{2}\mathrm{Hb}$, BOLD changes, and ERD/ERS in the alpha and beta bands during a motor task were analyzed. In the patients group, TD-fNIRS and fMRI data were highly correlated. TD-fNIRS and fMRI showed smaller hemodynamic changes and minimal or absent postmovement beta rebound in the patients group versus controls.

#### 3.2.4 Surgical

fNIRS–EEG is suited for intraoperative monitoring for its lightweight properties, particularly when few-channel systems are employed. Combined measurements have been applied for monitoring brain function and oxygenation during carotid endarterectomy (CEA) under general anesthesia,^144^^–^^149^ always employing a flexible one or a few channels fNIRS systems. The monitoring goal was to predict perioperative cerebral ischemia that required arterial shunting.^144^ In fact, clamping during CEA causes major changes in cerebral blood flow that can lead to brain insult.

${\mathrm{rSO}}_{2}$ measured using fNIRS as well as EEG signals were generally monitored bilaterally during carotid CEA. de Letter et al.^145^ aimed at evaluating sensitivity and specificity of fNIRS cerebral oximetry to ischemia during CEA using EEG as a gold standard. The cross-clamping changes of cerebrovascular ${\mathrm{rSO}}_{2}$ were compared with data from EEG analysis. A sensitivity of 100% and specificity of 44% were achieved at a cut-off value of 5% decrease in ${\mathrm{rSO}}_{2}$. They concluded that the fNIRS ${\mathrm{rSO}}_{2}$ metric alone was not sufficient for good procedure specificity and further validation was required. The same approach and similar results were investigated by other groups.^146^^–^^148^ Moritz et al.^148^ combined fNIRS–EEG with transcranial Doppler (TCD) and internal carotid pressure (stump pressure, SP) measurements. The EEG measurement relied on SEP estimates. In the 48 patients undergoing carotid surgery during regional anesthesia, cerebral ischemia was assumed when neurologic deterioration occurred. During clamping, the minimum ${\mathrm{rSO}}_{2}$, its percentage change, the percent changes of SEP amplitude, the mean TCD velocity, its percentage change, and the mean SP were recorded. Data analysis highlighted a best performance for TCD and ${\mathrm{rSO}}_{2}$ percent change and SP measurement. Lower performance was found for SEP monitoring. Mauermann et al.^146^ investigated 90 patients undergoing unilateral CEA with bilateral fNIRS–EEG. Changes in cerebral ${\mathrm{rSO}}_{2}$ were assessed. A general decrease in ${\mathrm{rSO}}_{2}$ during carotid cross-clamping for CEA was associated with EEG-determined need for shunting. Pennekamp et al.^147^ performed both fNIRS–EEG and TCD measurements in a prospective cohort study. An intraluminal shunt was placed selectively determined by predefined EEG changes in alpha, beta, theta, or delta activity. ${\mathrm{rSO}}_{2}$ in the frontal lobe and mean blood flow velocity (from TCD) were estimated. An ROC analysis revealed a threshold of 16% decrease in ${\mathrm{rSO}}_{2}$ and 48% decrease in mean velocity as the optimal cut-off value to detect cerebral ischemia during CEA under general anesthesia. The authors found moderate sensitivity but very high specificity of both fNIRS and TCD measurements. They suggested that fNIRS measurements could be suitable for excluding patients from unnecessary shunt use. Perez et al.^149^ found that the surgical-side and contralateral-side ${\mathrm{rSO}}_{2}$ dropped significantly below the baseline values during clamping (−17.6% and −9.4%, respectively). After shunting, the contralateral-side ${\mathrm{rSO}}_{2}$ returned to baseline while the surgical-side ${\mathrm{rSO}}_{2}$ remained significantly below baseline (−9.0%) until the shunt was removed following surgery. At clamping, the surgical-side and contralateral-side processed EEG also dropped below baseline (−19.9% and −20.6%, respectively). However, following shunt activation, the processed EEG returned bilaterally to baseline values. During the course of this research, they found the ${\mathrm{rSO}}_{2}$ monitor to be clinically more robust (4.4% failure rate) than the processed EEG monitor (20.0% failure rate). The authors concluded that fNIRS-based cerebral oximetry discriminates between the surgical and contralateral sides during surgery better than a stand-alone EEG. They also highlighted the possibility of integrated metrics for prediction of cerebral ischemia during CEA to decide whether to perform a carotid artery shunting.

fNIRS–EEG systems have also been applied for monitoring of cardiac surgery. In fact, various studies have demonstrated that many patients undergoing cardiac surgery have evidence of central nervous system suffering. Although evidence is compelling that cerebral emboli are a major cause of perioperative central nervous system morbidity in such patients, alterations in cerebral perfusion pressure and blood flow can also influence the extent of injury after an embolic insult.^150^

Nollert et al.^151^ evaluated 41 patients undergoing cardiac operations with extracorporeal circulation with fNIRS–EEG. HHb and $O_{2}\mathrm{Hb}$ were estimated with fNIRS. Neuropsychological testing such as the minimental-state test indicated reversible postoperative neuropsychological deficits in four patients. HHb and $O_{2}\mathrm{Hb}$ concentration changes in these patients supported the hypothesis that neuropsychological deficits in patients after cardiac surgery can be caused by intraoperative cerebral hypoxia.

fNIRS–EEG also found application for monitoring brain function and oxygenation during aortic arch repair in hypothermia or normothermia^152^ and arthroscopic shoulder surgery.^153^

#### 3.2.5 Psychiatric

fNIRS–EEG measurements were employed for psychiatric disorder assessment. An fNIRS–EEG recording can be helpful for studying a psychiatric population in natural settings. Neural correlates of bipolar disorder, schizophrenia, and game addiction were investigated.

fNIRS studies on bipolar patients indicated low frontal activity during a verbal fluency task and altered fNIRS responses compared with those of patients with major depressive disorder or healthy subjects. EEG highlighted altered gamma, beta, and alpha band activities always related to deficits of frontal activity and frontotemporal-parietal connectivity.^154^

Possible neurophysiological markers of language perception in schizophrenia were investigated using fNIRS–EEG recordings.^155^ In particular, fNIRS was coupled with ERPs that were previously proven to be a useful tool for studying language processing abilities in psychiatric patients. Twenty-two patients were exposed to sentences that were either literal, metaphoric, or meaningless. EEG analysis showed that both N400 and left-hemispheric activation were altered in psychiatric patients. Differently from controls, correlation analyses showed a poor metaphor-related fNIRS-ERPs relation in the psychiatric patients.

Lastly, an fNIRS–EEG investigation was conducted on a game-addicted Japanese population during game play.^156^ The recording showed a decrease in $O_{2}\mathrm{Hb}$ during game play correlated with a decrease in beta-band power but unaltered alpha band power in addicted subjects.

Although only a few studies were performed, combined fNIRS–EEG measurements are feasible for psychiatric patient evaluation.

#### 3.2.6 Rehabilitation

Rehabilitation is a fundamental part of postacute care in neurological disease. fNIRS–EEG monitoring of brain activity during recovery can be of great help in providing brain function information without restricting the patient movement. fNIRS–EEG monitoring has been applied for rehabilitation purposes, either for monitoring functional brain recovery^157^ or through procedures that rely on the previously described BCI technology, mainly dedicated to gait rehabilitation.^158^ Pittaccio et al.^159^ investigated the potential role of early passive motion in stimulating cortical areas of the brain devoted to the control of the lower limbs. They monitored brain activity in four volunteers over motor and somatosensory areas during active and passive mobilizations of the lower limbs by an fNIRS–EEG (64 channels-sensors) system. Spatial correlation analysis of recording procedures highlighted similar patterns of activity between active and passive mobilizations for both measurements, particularly in the contralateral premotor areas. The results suggested that passive motion could provide somatosensory afferences that are processed in a similar manner as for voluntary control. fNIRS–EEG has also been employed through BCI for postacute neurological motor recovery. BCI for stroke motor recovery includes intensive training linking brain activity related to patient’s intention to move the paretic limb with the contingent sensory feedback of the paretic limb movement guided by assistive devices. BCI training was demonstrated in a controlled study to significantly improve motor performance in stroke patients with severe paresis.^160^

## 4 Combined fNIRS–EEG: Current Limitations and Future Directions

fNIRS–EEG systems exploit the ability of the two technologies to be conducted in conditions not suited for other neuroimaging modalities, and the multimodal approach is a useful tool for assessing brain electrical activity and hemodynamic/oxygenation state. A further development of the multimodal technology can be expected based on the constant positive trend over time and multiple fields of application of fNIRS–EEG scientific production within the last 30 years.

Among the limitations with respect to other techniques, such as fMRI, a poor sensitivity of fNIRS–EEG measurements to brain activity in deep brain cortex layers should be mentioned.^161^ Moreover, the main limitation when combining the two modalities derives from shared scalp surface and the presence of wires/fibers. In fact, poor mechanical reliability of sensors and/or optodes and lack of available scalp surface are possible problems that can be encountered during fNIRS–EEG integration, especially if the number of sensors used is large. Whereas EEG normally requires the use of gels, saline solutions, and/or pastes to keep the electrodes in place and to reduce impedance, a good fNIRS signal relies on mechanical rigidity and proper optode coupling with the scalp surface. Indeed, standard fNIRS inject and detect light in and from the scalp through optical fibers/fiber bundles. Optical fibers allow the subject to be electrically isolated. However, the fibers, combined with EEG electrical wires, may create discomfort to the patient, thus losing the lightweight, portability, and flexibility properties of the modalities. In fact, standard fNIRS detectors are difficult to be placed directly on the patient’s head, especially in the presence of conductive EEG gel.^162^ This is because of the high-voltages biases of the sensitive detectors employed, such as photomultiplier tubes or avalanche photo diodes. This practical disadvantage also introduces a long preparation time to place electrodes and optodes on the subject head. Although this limitation can be partially overcome by increasing mechanical stability and reliability of optical patches and electrodes caps, both systems require precise placement of dozens of electrodes/optodes on the subject scalp. Different procedures and technological advancements can be implemented to overpass these limitations. From the fNIRS standpoint, the use of light-emitting diodes, silicon photomultipliers (SiPM),^163^ and/or silicon photodiodes (SPDs) allows for increased space availability and the use of optical fibers/bundles to be avoided. The use of dry electrodes in EEG may allow avoidance of the conductive paste.^164^ It is worth mentioning that very few attempts have been made to create combined fNIRS–EEG solutions,^162^^,^^165^ and the two modalities have been implemented extensively using two separate wireline systems. To the best of our knowledge, there is lack of available solution on the market for stable and comfortable fNIRS–EEG helmets. Although advances have been made in the past 10 years, a perfect solution is not yet available, and most research teams build their own helmet. This aspect was clear when considering the great variability of the number of optodes and electrodes employed in the studies reported in this review. This issue is a clear limiting factor for a wide usage of fNIRS–EEG recordings in both clinical and nonclinical settings.

*Ad hoc* development of mechanical structures for an integrated system would allow for a stable and easy to wear fNIRS and EEG sensors layout. The development of portable, fiberless, and especially wireless combo fNIRS–EEG devices may extend the system application to everyday life settings. This is essential for the diagnosis of brain functional abnormalities as the artificial and tightly constrained settings may interfere with patients’ behavior and physiological status. Moreover, a wireless system is definitely more suitable in a clinical setting.

## 5 Conclusion

In this review, fNIRS, EEG, and combined fNIRS–EEG have been described and discussed. Moreover, major areas where multimodal investigation was employed both in clinical and nonclinical settings were presented. fNIRS, relying on spectroscopic measurements, can provide estimates of hemodynamic fluctuations and oxygenation of the brain, whereas EEG can be effectively used to measure the temporal dynamics of brain electrical activity. Thus, fNIRS and EEG provide different physical and physiological information, encouraging their flexible and lightweight integration in multiple brain research fields.

Within nonclinical applications, fNIRS–EEG exploited the spatiotemporal hemodynamic and electrical evolution of the human brain functions. Studies were performed assessing brain responses to external sensory stimulation (auditory or visual),^69^^–^^71^ or more complex brain functions such as language,^80^ motor intention,^74^^,^^75^ WM,^79^ and emotions.^82^ fNIRS–EEG was also applied for studying social interaction.^85^^,^^86^ An interesting application in the brain function field was the study of brain state proprioception during changes of gravity conditions.^76^^,^^77^ Moreover, BCI clearly showed the beneficial effects of multimodal fNIRS–EEG recordings. Multimodal BCI found a main application in sensorimotor imagery^48^^–^^51^ and SSVEP classification.^53^ Neurovascular coupling was also extensively studied using fNIRS–EEG systems relying on the high hemodynamic information content of fNIRS. Neurovascular coupling was assessed with fNIRS–EEG using three different experimental approaches. Some groups^56^^–^^59^ studied neurovascular coupling relying on resting state paradigm. Others^8^^,^^60^ assessed neurovascular coupling based on task-related response. Neurovascular coupling relying on external vascular^63^^,^^64^ or electrical manipulation (through anodal tDCS) was further investigated.^65^^–^^67^ A minor field of application of fNIRS–EEG in a nonclinical setting was sleep research.^87^

Within the clinical environment, fNIRS–EEG was extensively applied to newborn monitoring due to its portability, lightweight properties, and adequacy for long-term monitoring. Generally, for newborn and for overall clinical research, sparser optical and electrical arrays were employed when compared with nonclinical applications. fNIRS–EEG have been applied for monitoring newborn cerebral hypoxia^106^^,^^107^ or newborn brain activity and oxygenation immediately after birth.^110^^–^^112^^,^^114^ Moreover, the fNIRS–EEG simultaneous monitoring was utilized during deep hypothermic circulatory arrest.^101^^,^^117^ Response to painful treatments received^118^ and degree of sensory-related brain responses was assessed.^121^^,^^122^ Another important application of fNIRS–EEG in clinical settings was epileptic patients monitoring. In this field, in similarity with the newborn one, fNIRS–EEG flexibility is essential for continuous recording. Many studies focused on the hemodynamic mechanisms before, during, and after seizures.^14^^,^^15^^,^^129^^–^^132^ Some studies were performed to improve the temporal detection of epileptic activity^134^ whereas others were directed toward epileptogenic focus^138^^–^^140^ identification. fNIRS–EEG was also applied for the study of status epilepticus with subtle manifestations in infants^142^ and for cortical myoclonous monitoring.^143^ The combined measurements have been also applied to monitoring brain function and oxygenation intraoperatively during CEA.^144^^–^^149^ Other intraoperative environments for fNIRS–EEG were cardiac surgery^150^ aortic arch repair in hypothermia or normothermia^152^ and arthroscopic shoulder surgery.^153^ Minor clinical applications were rehabilitation,^159^ children development (mental retardation,^123^ ADHD,^124^ and TBI^125^), and psychiatry (bipolar disorder,^154^ schizophrenia,^155^ and game addiction^156^).

Current limiting factors for the multimodal technology are the presence of optical fibers for fNIRS, the presence of conductive gel/pastes for EEG, and the absence of standardized helmets for the combined technology. Future technical trends for fNIRS–EEG may fully exploit the combined system flexibility and portability for clinical and nonclinical environments through the usage of high sensitive semiconductor light detectors (such as SiPM) located directly on the scalp for fNIRS and dry active electrodes for EEG in a portable, fiberless, and even wireless devices with standardized helmets, overpassing current limitations.
